# Prenatal and Early Postnatal Behavioural Programming in Laying Hens, With Possible Implications for the Development of Injurious Pecking

**DOI:** 10.3389/fvets.2021.678500

**Published:** 2021-07-16

**Authors:** Elske N. De Haas, Ruth C. Newberry, Joanne Edgar, Anja B. Riber, Inma Estevez, Valentina Ferrante, Carlos E. Hernandez, Joergen B. Kjaer, Sezen Ozkan, Ivan Dimitrov, T. Bas Rodenburg, Andrew M. Janczak

**Affiliations:** ^1^Division of Animals in Science and Society, Faculty of Veterinary Medicine, Utrecht University, Utrecht, Netherlands; ^2^Flanders Research Institute for Agriculture, Fisheries, and Food, Melle, Belgium; ^3^Department of Animal and Aquacultural Sciences, Faculty of Biosciences, Norwegian University of Life Sciences, Ås, Norway; ^4^Bristol Veterinary School, University of Bristol, Langford, United Kingdom; ^5^Aarhus University, Department of Animal Science, Tjele, Denmark; ^6^Department of Animal Production, Neiker, Vitoria-Gasteiz, Spain; ^7^IKERBASQUE, Basque Foundation for Science, Bilbao, Spain; ^8^Department of Environmental Science and Policy, Università degli Studi di Milano, Milan, Italy; ^9^Department of Animal Nutrition and Management, Swedish University of Agricultural Sciences, Uppsala, Sweden; ^10^Institute of Animal Welfare and Animal Husbandry, Friedrich-Loeffler-Institut, Celle, Germany; ^11^Department of Animal Science, Faculty of Agriculture, Ege University, Izmir, Turkey; ^12^Agricultural Institute - Stara Zagora, Stara Zagora, Bulgaria; ^13^Department of Production Animal Clinical Sciences, Norwegian University of Life Sciences, Oslo, Norway

**Keywords:** laying hen chicken, injurious pecking, behavioural programming, prenatal, epigenetics, incubation, early life development

## Abstract

Injurious pecking (IP) represents a serious concern for the welfare of laying hens (*Gallus gallus domesticus*). The risk of IP among hens with intact beaks in cage-free housing prompts a need for solutions based on an understanding of underlying mechanisms. In this review, we explore how behavioural programming *via* prenatal and early postnatal environmental conditions could influence the development of IP in laying hens. The possible roles of early life adversity and mismatch between early life programming and subsequent environmental conditions are considered. We review the role of maternal stress, egg conditions, incubation settings (temperature, light, sound, odour) and chick brooding conditions on behavioural programming that could be linked to IP. Brain and behavioural development can be programmed by prenatal and postnatal environmental conditions, which if suboptimal could lead to a tendency to develop IP later in life, as we illustrate with a Jenga tower that could fall over if not built solidly. If so, steps taken to optimise the environmental conditions of previous generations and incubation conditions, reduce stress around hatching, and guide the early learning of chicks will aid in prevention of IP in commercial laying hen flocks.

## The Problem of Injurious Pecking in Laying Hen Chickens

Ethical concerns among consumers in many countries have prompted a move toward housing systems that take the behavioural needs of farm animals into account. This has led to a ban on conventional battery cages for laying hens in Europe (1). In other parts of the world, a similar transition is happening where battery cages are replaced by furnished cages or cage-free systems with or without outdoor access. In cage-free systems, hens have greater behavioural opportunities and freedom of movement but, when management is not tuned to behavioural programming in these systems, there is greater risk that injurious pecking (IP) will develop as compared to cages (2).

**Figure 1:**
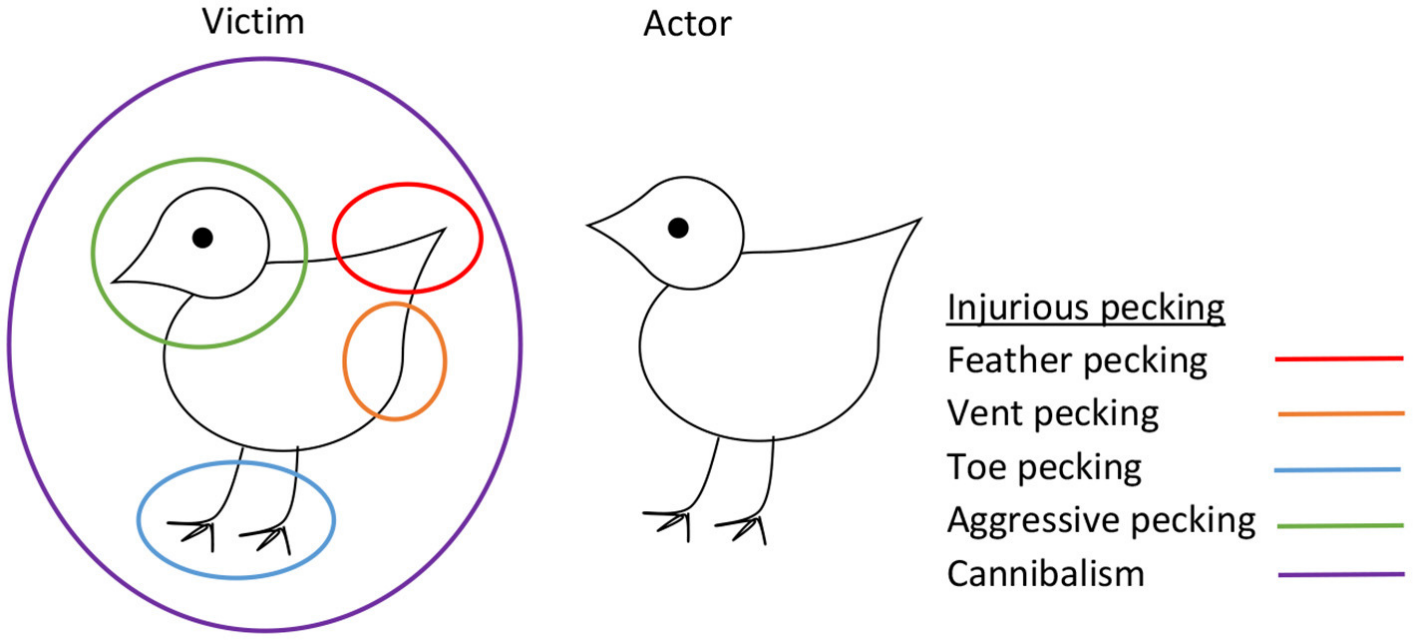
Injurious pecking in laying hens, showing different types of pecking in relation to target areas on the victim.

IP refers to damaging bird-to-bird pecking whereby pecks to the feathers or tissue of another bird cause plumage damage, skin wounds, or tissue damage. IP includes severe feather pecking (SFP), vent (i.e., cloacal) pecking, toe pecking, and aggressive pecking toward the comb, head, and neck of the recipient (3) (Figure 1). Severe injuries can lead to cannibalism and death of the victim. Collectively, these different forms of IP represent an important welfare and economic problem, especially in chickens selected for high egg production. For example, SFP (whereby feathers are damaged or removed) has been reported to affect more than 50% of layer flocks in various European countries (4–8).

Beak treatment (the removal of the sharp tip of the beak) is the most common method to reduce the impact of IP. Infrared or hot blade treatment is typically performed at hatch or before day 10 of age. Both treatments cause pain (9, 10) and can impair beak-related activities (11), such as eating, drinking, and removal of ectoparasites (12). For these reasons, several EU countries have banned or omitted beak treatment. In hens without beak treatment living in cage-free housing systems, IP can potentially result in thousands of victims being damaged and result in many casualties both during rearing and in adulthood, thus compromising hen welfare (13). Therefore, the egg industry is urgently seeking solutions that prevent and control IP. To avoid poor hen welfare due to IP, preventive solutions are preferable over curative solutions.

There is a wealth of published literature on risk factors for the development of IP during rearing and the laying phase, revealing that IP is a complex and multifactorial problem (14, 15). However, the involvement of prehatch and early post-hatch factors on the development of IP has been far less studied. In both mammals and birds, the embryonic environment can exert strong and long-lasting effects on offspring behaviour. Hormonal and epigenetic mechanisms that prepare the developing embryo for its future environment can be maladaptive when postnatal conditions differ from the parental conditions (i.e., a mismatch) (16–18). Here, we review how behavioural programming *via* prenatal and early postnatal conditions could influence the development of IP in laying hens.

### Description of IP in Relation to Behavioural Programming Effects

A distinction is made between pecking arising from aggressive and non-aggressive motivation, as the body targets and post-hatch risk factors differ (19). Non-aggressive IP is considered a redirected form of foraging behaviour, as both pecking during feeding and IP show similar motor patterns (20). Preferences for pecking at feed particles in substrates are established early in life (21). Innate sensory and motivational mechanisms prompt newly hatched chicks to peck at or avoid certain objects, forms, colours, and shapes (22). In natural settings, the mother hen's behaviour supplements this individual learning with more explicit information about what to peck, thereby increasing the valence of desirable pecking targets (23–25). Under commercial conditions where chicks are reared in the absence of their mother's guidance, direction of foraging pecks toward flock mates could result from a chick's failure to learn to direct these pecks toward appropriate substrates and feed items. In addition, absence of suitable manipulable foraging material can lead to IP in chicks (26–29).

SFP can, nevertheless, co-occur with pecking in the litter (28, 29). An association has been found between a high occurrence of litter-directed pecks by individuals when young and a high level of SFP and litter-directed pecks when adult (13). This suggests that SFP is not a direct substitute for foraging when a substrate is available, but that some individuals have a high pecking motivation overall and are thereby more prone to develop IP (30). For example, in a genetic line selected for high levels of SFP and non-damaging allo-pecking such as gentle feather pecking (GFP), neurological changes were associated with hyperactivity (31, 32). Specifically, these laying hen lines showed dysregulation of the serotonergic and dopaminergic systems (33). These findings are significant because dopamine and serotonin (5-HT) are important modulators involved in the regulation and motivation of feeding behaviour, aggression, impulsiveness, and reward systems [see de Haas and van der Eijk (33) for the role of 5-HT in IP] and dopaminergic and serotonergic activity can be influenced by prenatal environmental conditions. Environmental conditions leading to maternal stress in mammals have been linked to altered brain function in offspring, including perturbations in neurotransmission, an overactive hypothalamus–pituitary–adrenal (HPA) axis, and increased susceptibility to impulsivity and anxiety (34). Anxiety (sustained fear in the absence of acute danger) is a state linked to alterations in serotonergic activity (35). In laying pullets, high anxiety levels and high levels of activity in a fear-evoking test situation have been related to IP as an adult (36, 37). We explain below how the aforementioned findings could be related to prenatal behavioural programming.

## Hypotheses About How Prenatal Behavioural Programming Could Influence IP

**Figure 2:**
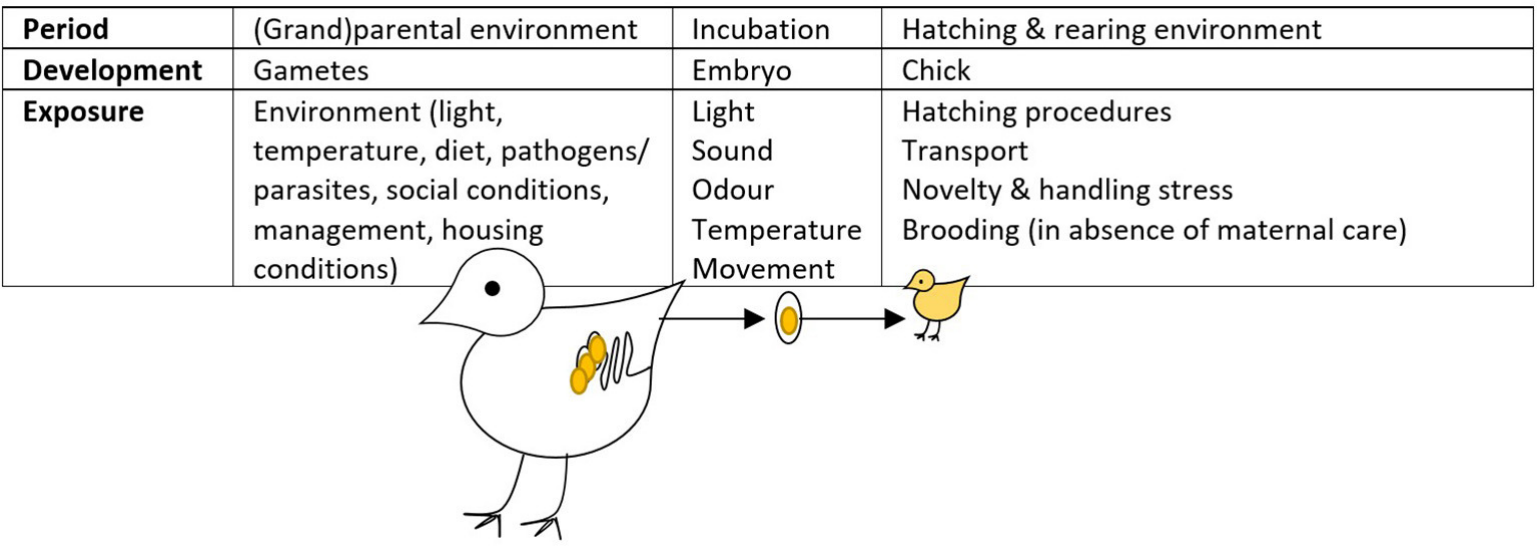
Periods where (embryonic) programming in the commercially-raised laying hen can occur [adapted from (39)]. This figure is amended from Buyse et al. (40) and Geurrero-Bosagna et al. (41).

Effects of the prenatal environment on the postnatal behaviour of animals have mostly been studied in the framework of embryonic programming. The term “embryonic programming” refers to the process by which exposure to certain stimuli during sensitive periods of embryonic development results in physiological, metabolic, and epigenetic changes with long-term implications (38). In birds, these sensitive periods occur during egg formation in the mother and, further, in the shelled egg where the embryo develops, and in early post-hatch life (Figure 2).

Various hypotheses about the biological significance of embryonic programming have been proposed. According to the “silver spoon” hypothesis, flavorable early life conditions result in individuals that grow better, with higher survival and reproductive success than those developing in a harsh environment (42). However, the benefits of “being born with a silver spoon in one's mouth” may depend on the environmental conditions subsequently encountered, and the animal's genetic predisposition. Based largely on human studies, the “Barker hypothesis” (43) proposed that exposure to adverse early life environments leads to metabolic and physiological changes that increase the risk of developing a disease when exposed to affluent living conditions during adulthood (44). While this hypothesis originated from observations based on the early postnatal period, it soon became evident that the embryonic environment could also play a role in determining the risk for disease during adult life. According to the “environmental matching” or “predictive adaptive response” hypothesis, responses to environmental conditions during embryonic development prepare the embryo to cope with the same conditions postnatally (45). Thus, the prenatal developmental responses would be adaptive if the environment remains stable following birth, but maladaptive in situations where there is a mismatch between the predicted and actual postnatal environment (46, 47).

Controlled experimental studies in sheep, guinea pigs, and rats have provided support for the idea that an adverse embryonic environment can induce postnatal physiological and metabolic alterations that increase the risk of developing metabolic disease. These include alterations in stress sensitivity *via* the HPA axis, hypertension, and obesity (48, 49). For example, rat offspring from mothers that were undernourished as opposed to well-nourished during pregnancy exhibited higher feed intake, obesity, hypertension, and reduced voluntary locomotor activity as adults (50). These effects were exacerbated when a high-fat diet was provided postnatally, heightening the mismatch between embryonic low and postnatal high nutrient availability (50). The resulting “thrifty” phenotype stores and conserves energy through hyperphagia, increased fat deposition and sedentary behaviour. This is thought to be advantageous in environments with low or irregular feed availability, but at the expense of increasing the risk of metabolic diseases in environments with high nutrient availability (50). There could be profound implications of these mechanisms for farmed animals such as laying hens, where discrepancies exist between the breeding and rearing environmental conditions that may predispose offspring toward IP.

### Maternal Effects in Relation to Behavioural Programming in Chickens

Several studies have investigated embryonic programming of behaviour in chickens in response to maternal stress (Table 1). These studies have manipulated environmental conditions experienced by mothers during hatching egg production and evaluated outcomes in their chicks. For example, in broiler chickens, mothers feed-restricted around conception (4 weeks before to 1 week after conception) had offspring that were smaller at hatch than offspring from mothers that were fed *ad libitum* around conception, although they caught up in weight by 6 weeks of age (woa) (51). Interestingly, the female offspring of feed-restricted mothers had a greater accumulation of abdominal fat at 6 woa, but only when fed *ad libitum*, supporting an effect of mismatch between the maternal and offspring environments. Maternal nutritional stress has also been manipulated in studies on laying hens. A higher ratio of omega-3 to omega-6 polyunsaturated fatty acids in the maternal diet was associated with increased faecal corticosterone metabolites (FCMs) in the hens and elevated fear of a novel object in their chicks (52). Thwarting hens' access to feed for 6 h daily at unpredictable times was also associated with elevated FCM in the hens and more fearful, less competitive adult female offspring (53).

**Table 1:** Examples of environmental effects on parents, their eggs, and their offspring in chickens.

**Parental treatment**	**Type of bird, age at treatment and duration**	**Effects on the parents**	**Effects on the eggs**	**Effects on yolk-hormone levels**	**Effects on development, behaviour and physiology of the offspring**	**Epigenetic effects**	**References**
Food restriction	Broiler breeder ♀ 60–65 woa for 6 wks	≈ Fertility	N.D.	N.D.	≈ Hatchability ↑ Hatch window ↓ Hatch weight, ↓ naval quality^♀^ ≈ BW, tibia length, % left ventricular weight ≈ Ascites incidence to 6 woa ↑ Abdominal fat^♀^ *^adlibitumfeed^* at 6 woa	N.D.	(1)
Diet high omega 3:6 ratio vs. high omega 6:3 ratio	WL ♀ adult for 6 wks	↑ FCM ≈ Food intake ≈ BW ≈ Laying rate	↓ Egg mass ↓ Yolk mass	↑ P ↑ A4 ↑ E2	↓ BW at hatch ≈ Growth rate to 1 woa ≈ Food neophobia at 4-5 doa ↑ Latency to eat, ↓ time eating at novel feeder at 6 doa	N.D.	(2)
Unpredictable access to food	WL♀♂ 26 woa for 11 d	↑ FCM ≈ BW	N.D.	≈ CORT	≈ BW at hatch ≈ Growth rate ↑ TI at 22 woa ↓ Food competition at 23 woa	N.D.	(3)
Unpredictable light-dark rhythm	WL, RJF ♀♂ 35–260 doa	↓ Spatial learning	N.D.	≈ CORT	↑ BW at hatch^RJF^ ↑ Growth rate to 8 doa ↑ Food competition at 21 doa ↓ Slower spatial learning^WL^ at 33 doa	Corr: Brain gene expr. parents and offspring	(4)
Unpredictable light-dark rhythm	WL ♀♂ 26 doa - adult	↑ Foraging pecks ↑ Pref. easy-found food ↑ Growth young^♀^ ≈ Food competition	N.D.	↑ E2 ≈ CORT ≈ A4 ≈ T ≈ DHT	↑ Pref. easily-found food at 55–57 doa ^♀^ ↑ Pref. high-energy food at 216 doa ↑ Food competition at 189 doa ≈ Food competition at 22 doa ↑ BW gain 66–105 doa ↑ Survival to 40 woa	Corr: Brain gene expr. parents and offspring	(5)
Moderately high ambient temp	WL ♀ 22–27 woa	↑ Body temp. ≈ CORT ≈ Feed intake ≈ TI ≈ Laying rate	↓ Egg mass ≈ % Yolk mass	↑ P ↑ T ↑ E2 ≈ CORT ≈ A4	↑ Chick quality score at hatch ↓ BW 0-20 doa ≈ Body temp. at 0 and 6 doa ≈ Feed intake to 3, 11, and 17 doa ↓ Pref. high-energy food at 10 doa ↓ Pref. sucrose solution at 21–22 doa ↓ Distress calls - novel food at 12 doa ≈ Latency to eat novel food at 12 doa ≈ TI at 7 doa ≈ Open field behaviour at 23–24 doa ≈ Adult hen BW and laying rate	N.D.	(6)
Unpredictable human movement, rough handling vs. predictable human movement, gentle handling	WL ♀ Adult for 5 wks	↓ Fertility ↓ Prox. to human ↑ Vigilance ↓ Feeding ↓ Exploring ↓ Resting ≈ FCM ≈ BW ≈ Laying rate	≈ Egg mass ≈ Egg comp	↓ P ↓ E2 ≈ A4 ≈ T	≈ Hatchability, BW hatch ≈ Pref. familiar vs. unfamiliar chicks at 20 doa ≈ Fear of human hand at 3 doa ≈ Fear of novel food at 8–9 doa ≈ Fear of NO at 8–9 doa ≈ Detour test behaviour at 10 doa ≈ Open field behaviour at 15–16 doa ≈ Social discrimination at 19 doa ≈ Social reinst. at 20 doa	N.D.	(7)
CORT implant	WL ♀ ISA Brown ♀ 33 woa Egg collection for 20 days	↑ CORT ↓ P, T, E2^WL^ ↓ Laying rate^WL^	↓ Egg mass ↓ Yolk mass ↓ Alb. mass + shell mass	↓ P ↓T ≈ E2	≈ Hatchability and sex ratio ↓ BW at hatch catch-up by 4 woa ↓ Food competition at 9 doa ≈ Time near NO at 11 doa ↓ Visual lat. with NO at 11 doa ≈ Open field behaviour at 14 doa ↓ TI at 28 doa and 24 woa ≈ CORT restraint at 10 woa ↑ Baseline T at 12 woa ≈ Baseline T at 24 woa ↓ Immune response at 12 woa	N.D.	(8)
On-farm variation: tests and analysis conducted on flock level/flock averages	Dekalb White ♀♂ ISA Brown ♀♂ 40 woa	Corr. ↑ fear NO ^*^↓ BW, egg mass ^*^ feed intake^DW^, ↑ Fear human ^*^↑ Mortality^ISA^	Corr: ↑ CORT ^*^ ↓ egg mass	N.D.	Corr^+^ ↑ CORT ^*^↑ SFP at 1 woa^DW^ ↑ 5-HT ^*^↑ SFP at 1 woa^DW^ ↑ F score ^*^↑ SFP at 1 woa^DW^ ↑ 5-HT ^*^↑ distress calls at 1 woa ↑ F score ^*^↑ distress calls at 1 woa ↑ F score ^*^↑ distress calls at 5 woa^DW^ N.A. parent stock ^*^ fear offspring N.A. parent stock ^*^ GFP offspring N.A. parent stock ^*^ feather damage N.A. parent stock ^*^ CORT or 5-HT	N.D.	(9)
Unpredictable stressors: isolation, cold ambient temperature, feed/water deprivation and handling	WL ♀♂ Daily during 4-26 doa	↑ BW adult^♀^ ↑ Assoc. learning^♀^ at 50–51 doa ↓ CORT restraint ≈ T^♂^ ≈ E2^♀^	↑ Egg mass^3of12d^	↑ T ↑ E2^1of3d^	↑ BW^♂^ at 56 & 74 doa ↓ BW^♀^ at 2 doa and ↑ BW at 74 doa ≈ Open field behaviour at 33–34 doa ≈ Social reinst. at 39-40 doa ≈ TI at 46-47 doa ↑ Assoc. learning response at 51-52 doa ≈ Correct assoc. learning at 51-52 doa ↓ CORT restraint^♂^	Corr^+^: Thalamus gene expr. parents and offspring	(10)
Repeated food frustration, restraint and social isolation	WL ♀♂ At 2 woa At 8 woa At 17 woa For 6 days	↓ Growth rate^2^ ↓ Vigilance NO^8^ ↓ TI^2, 8^ ↑ CORT restraint at 29 woa^8^ ≈ Emerg. at 30 woa ≈ Rec. restraint ≈ TI at 31–32 woa	↑ Egg mass ^2>17andC^	N.D.	↑ BW hatch^17vs.C^ ↑ BW at 4 woa^8vs. 2^ ≈ BW at 11 doa and 8 woa ≈ Open field behaviour at 11 doa ≈ TI at 18 doa ↑ Emerg. at 23–24 doa^17vs.C^ ↑ CORT restraint at 7 woa^8vs.C^	Corr: Brain gene expr. parents^2, 8, 17^ and offspring at 7 woa	(11)
Unpredictable stressors: simulated predator, air horn, unfamiliar conspecific, restraint, crating, transport	5 strains adult ♀: Brown 1 and 2, White 1 and 2, WL Daily for 8 d at 32, 52, and 72 woa	↑ CORT (acute) ≈ Basal CORT	N.D.	N.D.	≈ Hatchability and sex ratio ↓ Late embryonic mortality^Brown 2 and White 2^ ≈ BW at 0–17 woa ↓ Distress calls at 5–10 doa^White 2^ ≈ TI at 9 woa ≈ CORT ≈ Fear responses	N.D.	(12)

*5-HT, whole blood serotonin; A4, androstenedione; alb, albumen; assoc, associative; BW, body weight; C, control; comp, composition; CORT, plasma corticosterone; corr, correlation; corr+, positive correlation; DHT, 5-alpha-dihydrotestosterone; d, days; doa, days of age; DW, Dekalb White hybrid; E2, oestradiol, emerg., emergence test (latency to emerge); FCM, Faecal CORT metabolites; F score, feather score (feather damage); GFP, gentle feather pecking; ISA, ISA Brown; lat, lateralisation; N.A., no association; N.D., not determined; NO, novel object; P, progesterone; pref, preference; prox., proximity; RJF, Red Junglefowl; rec., recovery; reinst., reinstatement; expr., (gene) expression; SFP, severe feather pecking; T, testosterone, TI, tonic immobility duration; temp, temperature; WL, White Leghorn; woa, weeks of age*.

*Superscripts: effects found in a specific strain, sex, age, or treatment group*.

*↓ Lower; ↑ higher; ≈ similar; ♀ hens*;

♀ *effect only in hens*;

*♂ cockerels*;

♂ *effect only in cockerels*;

* with (e.g., association a*b).

*1: van der Waaij et al. (51); 2: De Haas et al. (52); 3: Janczak et al. (53); 4: Lindqvist et al. (54); 5: Nätt et al. (55); 6: Bertin et al. (56); 7: Bertin et al. (57); 8: Henriksen et al. (58); Henriksen et al. (59); 9: de Haas et al. (60); de Haas et al. (28); 10: Goerlich et al. (61); 11: Ericsson et al. (62); 12: Peixoto et al. (63); Peixoto et al. (64)*.

The influence of maternal stress on laying hen offspring is further supported by findings from studies imposing unpredictable lighting, which probably also affects the hen's nutritional status. Unpredictable lighting impaired spatial learning in both parents and their offspring and increased early growth rate and competitiveness of the offspring (54). While these effects were found only in offspring of a commercial hybrid [White Leghorn (WL)] and not the Red Junglefowl, the latter's chicks were heavier at hatch. In another study imposing an unpredictable light–dark rhythm (55), the offspring of stressed hens had faster growth and were more likely to select freely available feed over preferred feed that was harder to find. The offspring were also more competitive over feed as adults and had higher long-term survival.

Other environmental manipulations have indicated effects of the maternal environment on offspring development. For example, high ambient temperature in the hen's environment resulted in chicks that were less likely to vocalise when presented with novel feed and showed reduced preferences for high-energy feed and sucrose solutions compared to chicks of non–heat-stressed hens (56). Hens exposed to negative (vs. positive) human behaviour (unannounced arrival, moving fast with waving arms, carrying hens upside down) had chicks that were less likely to distinguish between familiar and unfamiliar conspecifics (57). Furthermore, the chicks of hens exposed to different acute stressors vocalised less when socially isolated, although only in one of five strains studied, namely, a specific White hybrid (63).

Implants that artificially increase the plasma corticosterone (CORT) levels of laying hens have been used to manipulate maternal physiological stress. CORT (vs. placebo) implants resulted in lower offspring fear and competitiveness and less lateralisation in visual inspection of a novel object (58). These effects were accompanied by lower egg mass and smaller chicks, suggesting that the behavioural effects could have been caused by prenatal undernutrition (58, 59).

To date, only one on-farm study has related maternal physiology in breeding flocks to offspring IP in rearing flocks (28, 60). A subset of hens from parental flocks was sampled for plasma CORT, whole-blood 5-HT, and feather damage. The average levels of fearfulness and SFP were related to average levels of behaviours assessed in their rearing flocks. Average levels of maternal CORT, 5-HT, and feather damage were positively related to the average SFP levels of the offspring at 1 woa. Maternal 5-HT levels and feather damage were also positively associated with distress calls of the chicks in a social isolation test at 1 woa. Moreover, maternal flock average CORT was negatively correlated with flock average egg mass (60). No maternal effects were found at 10 or 15 woa, and offspring could not be further assessed in adulthood. Interestingly, the link between physiology of the hens and behaviour of the chicks was seen only in a commercial WL hybrid and not in a Rhode Island Red (brown) hybrid (28). This strain difference is consistent with the strain differences reported in other studies (54, 63), as well as the finding of a higher proportion of methylated genes in response to an unpredictable light schedule in the brains of a WL commercial hybrid than in Red Junglefowl brains (65). Thus, the prenatal environment can have different effects depending on genotype.

**Figure 3:**
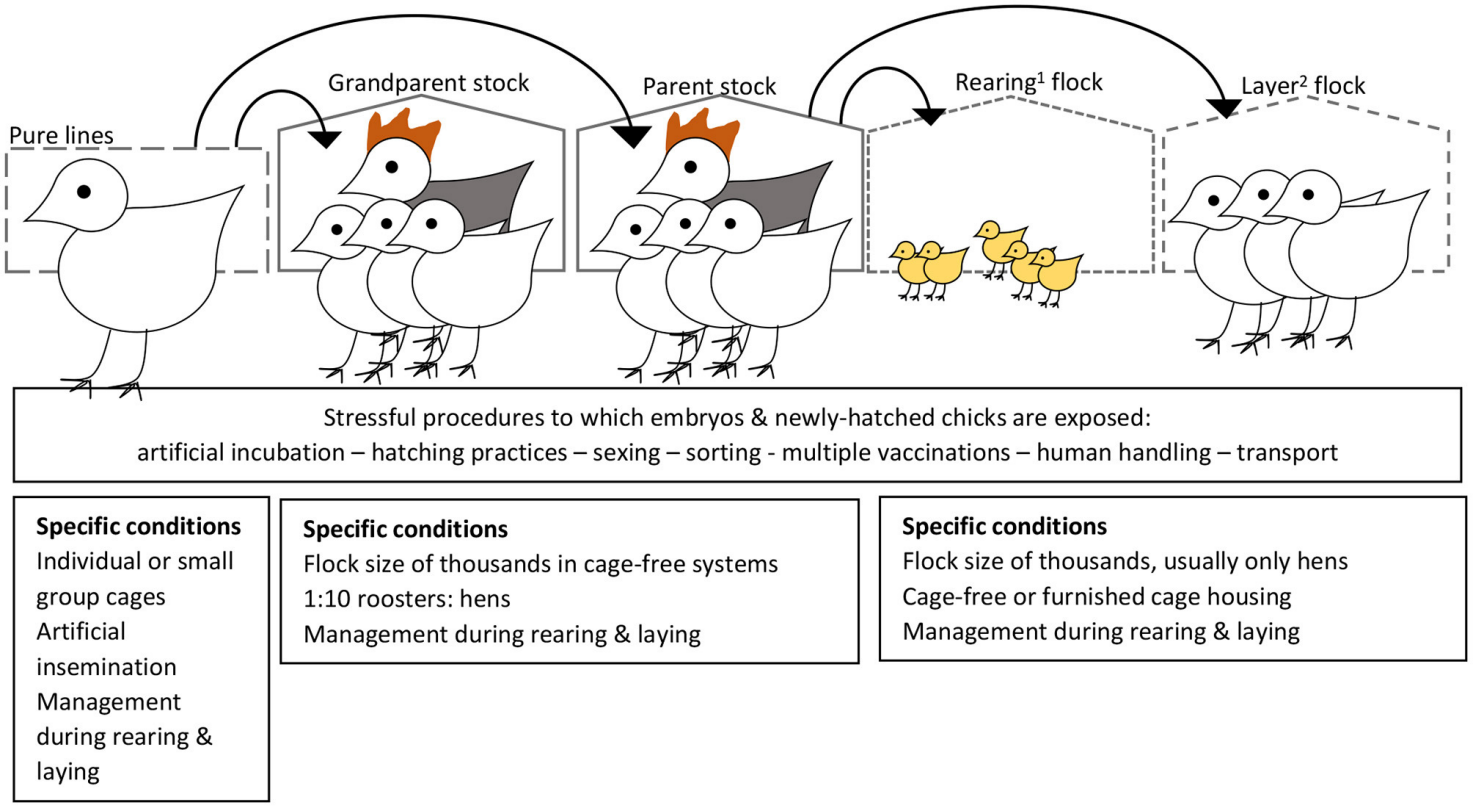
Set-up of the commercial egg-industry, and sources of variation in environmental conditions within and across generations that could lead to differential embryonic programming of behavioural phenotypes and subsequent risk for development of injurious pecking behaviour. ^1^Rearing of pullets occurs from arrival from the hatchery (referred to as 1 day of age) until ~17–18 weeks of age. ^2^The point-of-lay hens are then transported to the laying house (typically at a different farm), where they are kept until the egg production rate and egg quality drop toward economically non-viable levels (typically 80–100 weeks of age). Although egg farmers would prefer pullets reared in a similar housing type (e.g., aviary vs. cage) with similar equipment set-up (e.g., feeder, drinker and perch types, and locations) to that at the laying farm, some variation is almost inevitable. Management of the rearing and laying flocks needs to coordinated (e.g., similar dietary ingredients and lighting schedule at the point of transition to the laying house) for a smooth, relatively low-stress transition. Risk factors for development of injurious pecking have separate as well as interacting effects across the rearing and laying phases (7, 8, 14, 28).

Some of the above studies applied treatments only during lay. These studies provide evidence that environmental conditions experienced by the mother during the laying period can affect embryo development, leading to postnatal differences in behaviour and physiology. These effects may be mediated by conditions in the egg (18). In studies where treatments commenced before maturation of the hens, early life maternal stress could have programmed the hens' stress susceptibility or had a direct effect on their gametes. Such long-term effects are of interest also because chicks kept for hatching egg production are exposed to a variety of stressful procedures in early life [e.g., handling at the hatchery to determine sex, transport from hatcher to conveyer belt to transport baskets, beak treatment (still practised in many countries), transport to the rearing farm, exposure to novel environments and often multiple vaccinations during early life; Figure 3]. In a study on newly hatched chicks, stress induced by hatchery processing practises was linked to IP in later life (66), raising the possibility that stress in young chickens could also affect their offspring *via* epigenetic mechanisms on their gametes.

Goerlich et al. (61) exposed parents of chickens to a chronic stress treatment at 4–26 days of life (Table 1). When subsequently tested, the females had enhanced cognitive behaviour in an associative learning task and a dampened stress response to restraint compared to controls. The early stressed hens and their chicks had some similar gene expression patterns in the thalamus/hypothalamus in response to restraint. In parents, corticotrophin-releasing hormone receptor 1 (males) and early growth response 1 were upregulated only after restraint and not at baseline. The authors suggested that *via* such stress-specific genes, the parental early life environment prepared the offspring for coping with stressful conditions. Ericsson et al. (62) applied multiple stressors to parents in different age periods (2, 8, and 17 woa). Different short- and long-term effects were found on the parents and their offspring, depending on the age period when stress was applied, with no particular sensitive period for all effects. However, stressors applied to parents at 8 woa showed the strongest effect on CORT responses of their chicks. These results provide support for transgenerational behavioural programming effects in chickens (39, 41, 67).

## Mechanisms Involved in Prenatal Programming in Chickens

### Epigenetic Modifications and Inheritance

IP shows erratic variation; population-wise, the average level of IP varies from hatch to hatch, and on an individual basis, phenotypes cover a broad scale (68, 69). This degree of variation suggests that non-additive genetic effects, such as dominance (one allele of the gene dominates the other allele), epistasis (one gene interacts with one or more other genes), and epigenetic programming (changes in the “epigenome” due to environmental influences), may play a role in IP. The epigenome responds to environmental inputs with a range of mechanisms that influence gene expression, including DNA methylation, histone modification, and nucleosome positioning (70, 71). A growing body of research on human diseases points to epigenetic factors being key players in the differentiation of cells during development and changes in gene expression pattern. Most epigenetic “mutations” (epialleles) are either neutral or deleterious, but in some cases, their responses to environmental challenges are adaptive (71). Usually, the epigenome is reset following fertilisation, removing the epigenetic signatures acquired during development or imposed by the environment. But some epigenetic changes persist across generations, with major implications for heredity, breeding, and evolution (67, 71).

In laying hens, early life stress was found to alter the expression of stress-related genes such as tryptophan 5-hydroxylase 2 and dopamine receptor D1A in the parents and their chicks (62). Treatment differences in gene expression were also correlated across generations in this study. These epigenetic modifications could either have been inherited (passed on from their parents *via* the germ cells forming the zygote) or acquired in the egg environment (41) (Figure 2). To date, studies are lacking on behaviour of the F3 progeny of stressed hens, which would support transgenerational inheritance of environmentally induced epigenetic changes in behaviour.

### The Egg: The Chick's Embryonic Environment

To understand when the embryo could be affected by maternal conditions, here we explain the development of a chick from gamete to oocyte and to embryo. A female chick hatches with all her gametes (3,000–4,000) in her ovary. At reproductive age, under the influence of high ovarian hormone activity (mostly estrogens), these oocytes begin to store layers of yolk in their cytoplasm. This process is called vitellogenesis and takes 7–10 days. Yolk nutrients for the oocyte come from the hen's diet *via* the digestive tract, metabolism in the liver, and passage through the blood. Yolk is the nutrient base for the embryo, containing mostly lipids, cholesterol, and vitamins A, B, and D, but also including gonadal steroid hormones. Only some cells become mature oocytes, whereas others fade away. Once an oocyte contains enough yolk to support embryo development, it is released from the ovary (i.e., ovulation) and undergoes meiosis. Ovulation causes contraction in the oviduct, which enables sperm stored in crypts in the lower oviduct to move to the upper oviduct where fertilisation takes place. Regardless of fertilisation, the oocyte continues down the oviduct, nurtured *via* capillaries of the thecal and follicular cells surrounding the oocyte. Over 24–28 h, albumin is added in layers followed by formation of the eggshell. Following oviposition, the shelled egg provides the embryonic environment. The chick develops in ~21 days during incubation by a broody hen or in an artificial incubator.

#### Egg Mass and Egg Composition as Affected by Maternal Conditions

The mass and composition of the egg (yolk and albumen mass, yolk hormones, and other constituents) varies between strains and is influenced by age, diet, and maternal stress (Table 1), thereby affecting embryo development. The hen's nutritional status (72) and diet (52) can influence egg mass, which is strongly correlated with chick mass. When a hen is stressed by (over)activation of her HPA axis, this could influence offspring development at the stage of gamete, follicle, or embryo, depending on when the stressor occurs and for how long it lasts, as well as how the hen adapts (Figure 2). As CORT is a metabolic and stress hormone involved in protein and lipid metabolism (18), elevated maternal CORT levels can impair egg formation. For example, heat-stressed hens kept at high ambient temperature had reduced egg mass compared to controls (48.2 vs. 50.7 g) (56), and de Haas et al. (52) observed reduced egg and yolk mass in nutritionally stressed hens. Artificial elevation of CORT with a CORT implant in hens led to higher plasma CORT in the hens and reduced egg mass, which resulted in smaller chicks (58, 59). Furthermore, commercial hens in parental flocks with higher basal levels of plasma CORT had eggs with lower weight both at peak and end of lay (60). These results suggest that (chronic) stress can have a detrimental effect on the egg nutrient supplies laid down for the embryo. In contrast, maternal stress prior to puberty tended to be associated with higher rather than lower egg mass (61, 65).

#### Yolk Hormones as Affected by Maternal Conditions

Variation in yolk hormones, especially testosterone (T), influences the behaviour of chicks (73). Within a clutch, egg yolk T varies per egg, which can result in chicks performing varying levels of begging and aggression (74). It is hypothesised that maternal androgens in avian eggs allow the hen to manipulate sibling competition within the clutch (74). In precocial species such as the chicken and quail, maternal conditions affect yolk progesterone (P: precursor of CORT), yolk androstenedione (precursor of T), yolk T [precursor of estradiol (E2)], and yolk E2. These maternal hormones are derived from cholesterol and can be subdivided into androgens (androstenedione, T, E2) and progestogens (P, CORT, aldosterone).

Environmental conditions can influence these yolk hormone concentrations in laying hens (75). Maternal heat stress was associated with higher yolk P, T, and E2 (56) and lack of habituation to humans with lowered yolk P and E2 (57) (Table 1). A high percentage of lipids in the maternal diet elevated yolk androstenedione, P and E2 (52). This dietary treatment also increased CORT metabolites in the faeces compared to control hens (52). Yolk androstenedione and E2 were also higher in floor housed hens compared to caged hens (75). Unpredictable light increased yolk E2 (55), and early life stress increased yolk T and E2 (61). However, elevating CORT in hens by an implant lowered yolk P and T, although not E2 (58). Yolk E2 is often elevated as compared to control conditions when hens are exposed to acute and chronic stressors. It has been argued that yolk E2 facilitates epigenetic modifications in chickens (65) and could thus be a driving force behind parental effects. Several studies report a lack of association between maternal stress and yolk CORT (53, 54, 56, 63), but artificially elevating egg CORT can have a negative effect on hatchability (64).

## Incubation Effects on Behavioural Programming in Chickens

**Figure 4:**
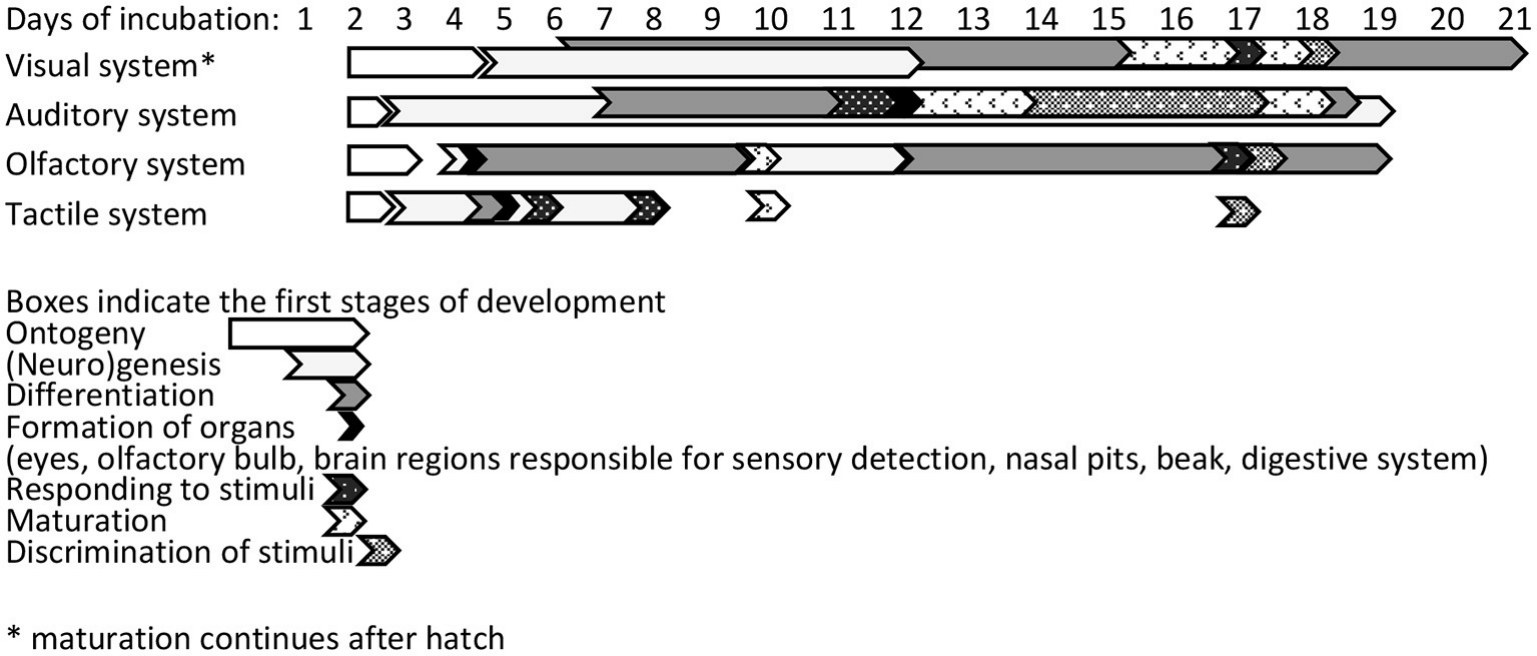
Timeline for sensory development of chicken embryos during incubation [based on (78)].

The ability of an embryo to respond to its environment during incubation and adjust its development is fundamental for its subsequent welfare and fitness (76). Developing chicken embryos sense photoperiodic, olfactory, and auditory cues in their environment and respond to them (77). Figure 4 illustrates the development of the visual, auditory, olfactory, and tactile systems during incubation.

### Effects of Light During Incubation

**Figure 5:**
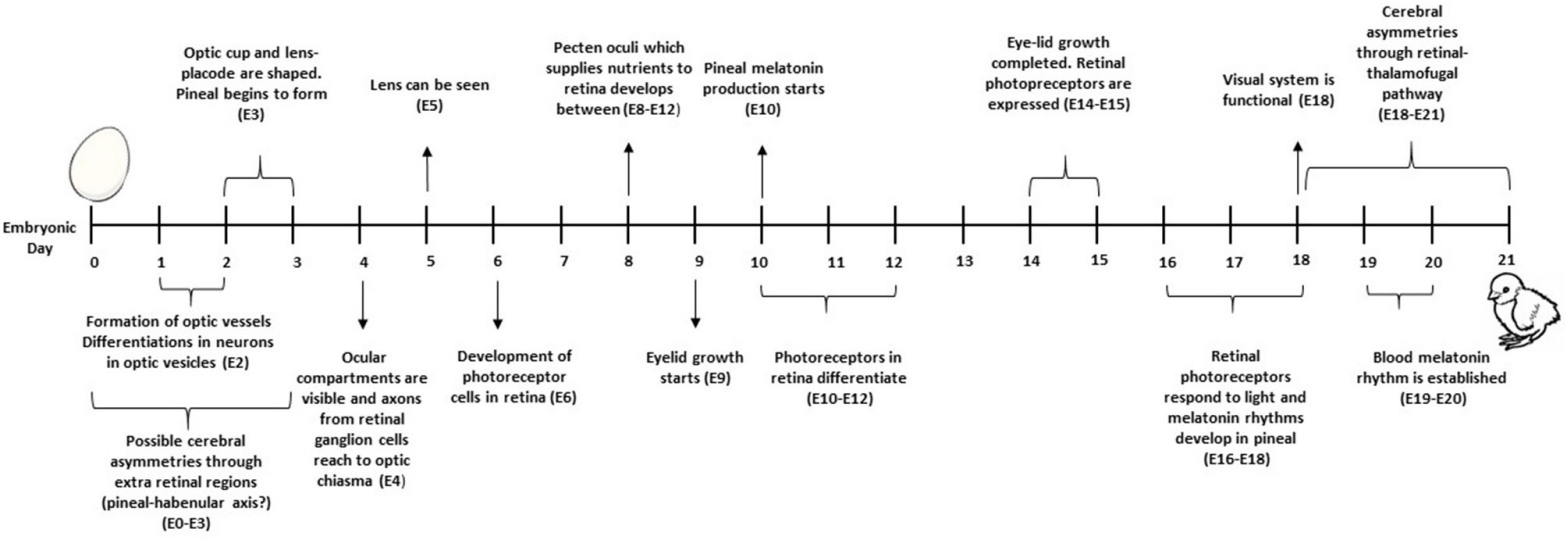
Timeline for development of the visual system.

Naturally brooded chick embryos receive short light pulses when the mother hen stands up or leaves the nest for feeding or drinking, or turns the eggs (79, 80). The undifferentiated neurons in the visual system of a chick embryo start to differentiate into neurons of optic vesicles on embryonic day (ED) 2, and axons from retinal ganglion cells reach the optic chiasma by ED4 (78) (Figure 5). The ocular compartments can be identified at ED4 (81). Eye formation is completed, and long-wavelength photo pigments are expressed, at ED14. The visual system is functional on ED18, but matures further post-hatch. From ED16, retinal photoreceptors respond to light/dark cycles through melatonin production (82). However, light-detecting photoreceptor cells are located not only in the retina, but also in the pineal and hypothalamus, and responses to light may start as early as ED3 *via* gene activation (83).

Chicken eggs are commonly incubated in darkness [24-h darkness (24D)]. Light during incubation (LDI) has been shown to modify the embryo's physiology and lead to changes in chicks' physiology and behaviour. A single pulse of 2 h of LDI at ED19 and ED20 led to higher GFP between 7- and 21-day-old chicks as compared to non-LDI chicks (84). This GFP was a form of social exploratory pecking whereby both familiar and unfamiliar chicks were targeted (84). Broiler chicks incubated with 16-h LDI and 8-h darkness (16L:8D) throughout the 21 days of incubation also performed more GFP, ground pecking, and preening at 5, 7, and 24 days of age compared to 0L:24D broiler chicks (85). However, in (male) layer chicks assessed at 3–7 days of age, GFP was unaffected by exposure of eggs to white fluorescent light from ED17–21 (86).

The wavelength of LDI appears to affect behaviour, physiology, and social pecking tendencies. A 16L:8D schedule from ED0–21 with green (520 nm) or white LED reduced fear at 3 woa in broiler chicks (85). Fear as assessed by vigilance was also reduced in layer chicks incubated in white light (87). White light increased GFP in line with the study by Riedstra and Groothuis (84) whereas green light reduced SFP and aggressive pecking in layer chicks (86).

LDI may have a positive effect on stress coping in chickens *via* early entrainment of a melatonin circadian rhythm (82). Focusing on behavioural effects, a clear day and night rhythm reduced fear at 3–6 woa in broiler chickens (88). Exposure to LDI in the last 3 days before hatching also improved discriminative learning (89) and spatial learning in feed-related tasks (90) in young layer chicks. These effects may originate from increased hemispheric lateralisation due to LDI. During incubation, the chick's left eye faces the yolk by ED18 and from ED18 is occluded by the body, whereas the right eye is always facing out and thus exposed to any light penetrating the egg shell (90). Exposure to LDI stimulates hemispheric lateralisation with positive effects on behaviour and welfare (91).

Lateralisation due to LDI improves cognition (92) and ability to perform the dual task of feed searching and predator detection (87), which increases foraging activity in nature (93). For example, chicks exposed to LDI used spatial and object-specific cues, whereas dark-incubated chickens used only object-specific cues (94). An increase in visual asymmetry *via* LDI can thus result in increased foraging efficiency. Chiandetti et al. (83) further suggested beneficial effects of LDI before development of the visual system, possibly due to changes in expression of genes related to photoreception. They incubated WL eggs in darkness or exposed to light either during the first 3 days or the last 3 days before hatching. Both groups of light-exposed chicks preferred the left side when attending to scattered feed grains and showed better spatial abilities. The relevance to development of IP lies in the possibility that LDI can improve feed discrimination in chickens. Given the link between foraging and IP, improved feed discrimination may lower the risk for development for IP.

### Effects of Temperature During Incubation

Eggs are incubated *via* skin conductance of the hen's broody patch (95). Development of a broody patch closely follows the onset of nesting behaviour (96). For domestic laying hens, a wide range of egg temperatures has been reported during natural incubation, with a minimum of 23.3°C and a maximum of 40.3°C (at air temperatures between 18.8 and 25.6°C) (96). Layer and broiler chicks used for production are incubated in large commercial incubators in which, for 18 days of incubation, ambient temperature is set within the narrow range of 37.5–37.8°C, and relative humidity (RH) to 55–60%. On ED18, eggs are transferred to a hatcher set at a temperature of 37.0°C with 65–75% RH. Temperature and RH are linked, thereby determining the effective temperature. Deviations from the optimal conditions of temperature and humidity may exert thermal stress on the embryos and consequently influence development and behaviour. Bertin et al. (97) exposed WL embryos daily to an ambient temperature of 27°C for 1 h twice per day during ED12 to ED19. This treatment resulted in higher fear in the chicks in a novel feed test and an open field test at days 8 and 14–15 of life, respectively. This increase in fear level was supported by an increased expression of corticotrophin-releasing factor (as assessed by fluorescent corticotrophin-releasing factor–positive cells) in the amygdala of treatment chicks vs. control chicks, a brain structure involved in the regulation of fear-related behaviour (35). No differences were found between treatment and control chicks in fear of novel objects or in social reinstatement behaviour (97).

Lay and Wilson (76) found that pullets exposed to 40.6°C for 24 h on ED16 had heavier adrenal glands at 11 woa when compared to controls, but did not differ from controls in basal or stress-induced CORT. In broilers, exposure to a continuous (24 h/d) or intermittent (12 h/d) temperature of 39.5°C from ED7 to ED16 resulted in higher plasma CORT at hatch compared to controls (98). Long-term adaptive effects were found when these birds were heat stressed at 35–36 days of life by exposing them to 35°C for 5 h. Birds incubated at the higher temperature had lower plasma CORT and mortality than controls (99). In wood ducks, incubation temperature had the opposite effect (100). Eggs were incubated at 35.0, 35.9, or 37.0°C, within the natural incubation temperature range. At 2 and 9 days of life, the ducklings incubated at the lowest temperature had higher baseline and stress-induced CORT compared to the ducklings incubated at the two higher temperatures. These findings show that manipulating temperature during incubation can affect postnatal HPA axis and stress responses in birds. Whether this stress coping strategy *via* temperature programming is linked to development of IP in laying hens is unknown.

### Auditory Imprinting During Incubation

Development of the auditory system begins around ED11 (101), and from ED16, the cochlea has developed the capacity of detecting and encoding sound (102) (Figure 4). Communication between the mother and developing chicks, and among chicks, starts the day before hatching (103). Vocalisations heard while still inside the egg are thought to help birds recognise their mother after hatching (103). During natural incubation, the embryos are also exposed to the range of sounds in their environment. In contrast, incubation in commercial incubators provides an acoustic environment different from that to which chickens are exposed to post-hatch. For example, during incubation, there is a constant background noise stemming from the motor and ventilation system. Tong et al. (104) reported this background noise to be 70 dB. Exposure to specific sounds has been shown to affect post-hatch social and cognitive behaviour of chicks. Sanyal et al. (105) tested one-day-old WL chicks in a T-maze, with a mirror at the junction to stimulate social reinstatement with companions in a brooder at the end of one arm. Exposure to arrhythmic noise of 110 dB from ED10 to hatch delayed latency to leave the start box and reach the brooder area (105). Furthermore, repeated testing did not improve spatial learning in these chicks. Kauser et al. (106) and Chaudhury et al. (107) subjected WL embryos to either no sound, species-specific sounds, or sitar music at 65 dB for 15 min/h on ED10 until hatch. All groups showed a decreased latency to reach the brooder over three trials (at 1, 2, and 4 days after hatch), but sound-exposed chicks were faster than non-exposed chicks. Music-exposed chicks were faster at day 1 of life, likely indicating reduced fearfulness. These studies indicate that sound during incubation can influence social behaviour, fearfulness, and cognition.

Naturally incubated layer chicks of a Swedish bantam were more likely than artificially incubated chicks to approach the clucking of a familiar hen (108). Similarly, one-day-old WL chicks exposed to sitar music (65 dB for 15 min/h) from ED10 until hatching responded more to species-specific maternal sounds than control chicks that received no auditory stimulation during incubation (109). Finally, the HPA axis, which plays a central role in controlling reactions to stress, was also affected by sounds during incubation. Sanyal et al. (105) found an increased concentration of plasma noradrenaline in day-old chicks that had been noise-stimulated as embryos compared to non-stimulated control chicks, with music-stimulated chicks being intermediate. No effects of treatment were found on the level of plasma CORT. In contrast, at 24 h post-hatch, Kauser et al. (106) found lower plasma CORT concentrations in chicks exposed to either species-specific sounds or sitar music during incubation compared to control chicks. At 72 h post-hatch, the plasma CORT concentration was higher in the species-specific sound-stimulated chicks. At 120 h of age, no difference in plasma CORT concentration was found between treatments. Rodenburg et al. (110) showed that exposure to noise during incubation delayed hatching and reduced vocalisation, but did not affect fearfulness at 5 woa in brown layer chicks. In summary, studies have shown that excessive noise during incubation influences fearfulness, cognitive abilities, social preferences, and the HPA axis in chickens, which could add to the risk of developing IP.

### Olfactory Conditioning During Incubation

Under natural conditions, chicken embryos develop inside a porous egg, which remains in close contact with the body of the brooding hen *via* the brooding patch. The egg is a porous matrix where gas exchange with the surrounding environment is possible. This environment entails the nest and nest material (i.e., bedding material, feathers, manure from the broody hen, feed particles). Odours from this environment can pass (to some extent) through the porous eggshell, exposing the developing chick to cues from its future environment. Scent or odours are chemical components that function as olfactory cues providing information about the environment. These odours can modify the receivers' behaviour (111). Chickens use odour cues to find their nest (112), avoid predators, and find food (113). Preen oil from the preen gland of chickens may have a specific odour or taste that is attractive to hens, as shown by ingesting specific feathers (114) and targeting IP to specific areas (115).

The chick's olfactory bulb evolves rapidly from the second day of incubation and is fully matured at hatch (78) (Figure 4). Developing chicks are able to discriminate different olfactory cues from ED10 of incubation (116). Chicks are more attracted to odours they have encountered during incubation than to unfamiliar odours. Porter and Picard (117) found that chicks exposed to the odour of oranges during the last 2 days of incubation (by placing eggs on odorised paper in the incubator) were faster to approach a container with the same odour 12 to 36 h post-hatch. Furthermore, olfactory cues during incubation can be used to influence feed preferences post-hatching; 4-day-old chicks that had received an olfactory cue at the end of incubation ate more feed with the same odour compared with control chicks (118). Thus, familiar odours to which chicks have been exposed during incubation can stimulate feeding. Sneddon et al. (119) exposed eggs to strawberry oil *via* different means during incubation (i.e., on the eggshell, in the air, or near the egg). Exposure to strawberry oil on the eggshell led to increased intake of strawberry-flavoured water, and more entries into a strawberry-scented environment, in the first days after hatching. This attraction to familiar odours is maintained throughout life (120).

As a practical strategy to avoid removal of familiar odours, disinfection of hatching eggs in the hatchery should be avoided. If eggs are disinfected, specific odours could be added during incubation, and exposure could be continued on the rearing farm. For example, Madec et al. (121–123) showed that broilers kept in an environment where an analogue of maternal hen's odour was provided had lower stress responses and enhanced growth and body weight up to 80 days of age, compared with unexposed broilers. It remains unknown whether odours have long-term effects on the development of IP. In the hatchery, eggs of different parental sources are often put together in the incubator. This practise could also lead to disease risks by contamination from one farm to another. Another practical strategy is to limit this contamination and reduce the need for disinfection by keeping the familiar odours from one farm and avoiding mixing eggs from different parental flocks during incubation.

## Early Post-Hatch Conditions and Behavioural Programming of Chickens in Relation to IP

Soon after hatching, chicks are able to react to different environmental stimuli with physiological and behavioural changes, often leading to long-lasting modifications in brain development referred to as developmental plasticity (124). Maturation of the brain *via* specialisation of brain circuits continues up to 10 woa (125). There are sensitive periods in which chickens learn certain behaviours, such as imprinting, when they develop strong preferences (25). Learned behaviour patterns can be programmed by early life conditions and prenatal modulations, resulting in a higher tendency to perform certain behaviours such as IP.

Feeding is one of the most important behaviours chicks need to learn to survive, along with learning to drink, find warmth, and hide from predators. As the chicken is a precocial species, the chick is not actively fed by the parents as in altricial species. However, under natural conditions, the mother hen guides the feeding behaviour of her chicks, with specific vocalisations to distinguish edible from inedible particles (25). Under commercial conditions, in the absence of a mother hen or older chicks, the newly hatched chicks need to find feed on their own, with the help of management practises. Chicks are strongly socially oriented and learn from each other what is edible. Thus, if IP occurs, the behaviour can spread rapidly through social learning (126, 127).

Housing and handling practises in the first few weeks post-hatch can have immediate as well as long-lasting consequences for IP (128). Hatching procedures (handling, sexing, conveying, transport) influence IP and stress sensitivity in later life (66). Rearing in cages or aviaries without a floor substrate to peck at is a risk factor for IP on rearing farms (28), an effect that persists in adults (129, 130) and is associated with epigenetic markers in red blood cells (i.e., 115 genomic changes in red blood cells from rearing in cages vs. in aviary) (131). Under natural conditions, the broody hen is a source of warmth and protection from predators, a function partly replaced by artificial brooders. Provision of dark brooders has been found to reduce the risk of IP as well as fearfulness in hens (132) and chicks (133).

## Discussion

As we have outlined, a myriad of prenatal and early postnatal factors can influence the behavioural development of poultry at different points in development, including prehatch and post-hatch housing and nutritional condition of the parental birds, the incubation environment, and egg and chick handling procedures. While there are clear indications that early post-hatch conditions play a role in the development of IP in poultry [e.g., see Hedlund et al. (66)], the influence of prehatch conditions on IP is currently unclear. Behavioural differences that could be associated with an elevated risk of IP (e.g., increased fear-related behaviour and competitiveness) have been detected, although in an inconsistent pattern across studies (Table 1). Differences in genetic strain and stress dose received by the embryo have likely contributed to these varied results. Whereas high levels of prenatal stress and exogenous CORT can be expected to impair embryo survival, as well as early chick competitiveness and growth, less extreme stress may program chicks with elevated early competitiveness and growth as a coping mechanism, although potentially at a cost of reduced longer-term health. Only one correlational study to date has specifically investigated associations between prenatal conditions and postnatal IP, indicating a positive association between elevated CORT and early postnatal SFP (60). There is thus good reason to pursue studies into the influence of embryonic stress on IP. Furthermore, the role of early (prepubertal) parental stress, and the possibility of transgenerational effects including those derived from the male lineage, should be pursued.

A better understanding of mechanisms involved in the development of IP is needed to improve our ability to predict and prevent its occurrence, especially under conditions or systems in which beak treatment is not carried out. While learning can be involved in the emergence of IP post-hatch [e.g., see Cloutier et al. (126)], the roles of neurogenesis and epigenetic changes are currently uncertain. These could operate in opposite directions whereby epigenetic marks induced by maternal stress increase the risk of IP whereas neurogenesis serves to enhance stress resilience (134), potentially reducing the risk of IP. Alas, investigating neurogenesis and epigenetic changes in behaviour-relevant brain regions requires sacrificing the animal. Finding relevant epigenetic biomarkers for IP risk factors in tissues that can be sampled repeatedly [e.g., red blood cells (131)] would aid longitudinal studies on the development of IP. In addition, the role of the gut microbiota in programming behaviour is an emerging topic, given its potential for influencing IP (135). There is evidence of microbial transfer from mother to offspring in chickens (136), raising questions about how maternal stress, egg handling practises, motherless chick rearing, and mismatch between the parental and offspring microbial environment affect establishment of a well-adapted microbiota in chicks and how these factors, in turn, alter the risk of IP.

The extent to which early life effects can be detected later is likely to be influenced by the degree of environmental adversity subsequently experienced. For example, nutritional imbalance, heat stress, pathogens, poor air and litter quality, lack of opportunities to engage in natural behaviours, and inability to avoid pecks by flock mates may override the effects of early life risk factors for IP. In contrast, early life conditions predisposing chicks to IP may be buffered and thus masked by flavorable environmental conditions experienced when older. For example, whereas placing newly hatched chicks in a cage with no substrate increased the risk for IP (28), environmental enrichment of the subsequent adult housing reduced the risk (129). Other possible buffering effects may be derived from the social support provided by the mother hen (137) and housing in a complex environment (138). These predicted effects raise a fundamental question about the role of a mismatch in environmental adversity. If the risk of IP is promoted by maternal stress resulting in programming of offspring to be thrifty, competitive, and anxious (i.e., prepared to survive adverse conditions in the most vulnerable early post-hatch period), IP may be exacerbated in a matching stressful post-hatch environment. If a mismatched, more flavorable post-hatch environment buffers this early programming, hens may be more prone to metabolic disorders such as fatty liver disease (139) as predicted by both the “Barker” and predictive adaptive response hypotheses. However, it is questionable whether the development of metabolic diseases in laying hens is associated with an elevated risk of IP. Likewise, it is unclear whether the predicted sociable, calm, adaptable offspring of parents reared in relatively flavorable conditions (i.e., prepared to live long in a bountiful environment) would be more prone to develop IP if subsequently finding themselves in a mismatched stressful environment.

Besides masking of early effects by later ones, identifying early life risk factors for IP is challenging for several additional reasons. Globally, most commercial flocks are beak treated, which reduces damage due to IP. Furthermore, a commercial flock may be derived from multiple parental flocks, and ready-to-lay pullets may be sourced from more than one rearing flock. Unless flock history is known, prehatch contributors to flock IP rates will remain hidden. Most but not all (60, 129, 130) studies into behavioural programming have therefore been based on randomised controlled experiments. However, the behavioural outcomes assessed have typically not included IP because it is often relatively rare and unpredictable under experimental conditions and involves harm that one is ethically bound to minimise. Long-term experiments are also expensive and labour-intensive. It is not surprising that studies done to date have typically involved relatively low sample sizes and have rarely extended from the parent generation to adulthood of the offspring or to subsequent generations. Studies focused on outcomes in young birds will fail to detect possible early organisational effects of yolk-derived gonadal steroids on IP that would only be activated at puberty.

A further challenge arises from our incomplete understanding of relationships between different forms of IP. Different forms of IP may obscure differential effects if they are pooled. For instance, cloacal cannibalism appearing following the onset of egg production is not clearly related to other forms of IP (140). Also, elevated aggressive pecking at the head may be negatively correlated with non-aggressive pecks at other body parts (19), but it is not always easy to distinguish between aggressive and non-aggressive, even playful, intent. It is recommended that future reports include data on the frequencies of different forms of IP, body region targeted, and the contexts in which pecks occur. Even if rates of IP are too low for statistical evaluation, these data could be subjected to meta-analysis in the future. Future studies might also explore the possibility that some aspects of aggressive and non-aggressive pecking, such as the frequency and force of pecks and thus risk of injury, are influenced by common epigenetic mechanisms.

Finally, group-level reporting of outcomes is a limitation of previous studies into early effects on IP because hens vary in personality and stress coping ability (141). Hens better able to command limited resources will be less affected by environmental adversity than those less able to maintain a place at the feeder, access a nest space in a timely manner prior to oviposition, avoid unwanted mating, or secure a nightly roosting spot in a preferred location. On the other hand, in a rich environment, highly competitive, anxious individuals may experience more stress than more tolerant, relaxed individuals. Within a flock, variation in the relative frequency of birds adhering to proactive and reactive stress coping styles could perhaps explain variation in results from different studies. This is relevant because IP depends not only on the behaviour of those that deliver potentially damaging pecks, but also on the responses of targeted receivers of pecks (140, 142). In addition, individual differences in maternal stress can be predicted to reduce flock uniformity in egg mass, hormone deposition in eggs, and growth of the offspring. The resulting stress could lead to asymmetric growth resulting in fluctuating asymmetry of bilateral morphological traits that increase the risk of receiving IP (143). Future use of computer imaging algorithms for individual tracking and automated behavioural analysis will facilitate evaluation of the contribution of different stress coping styles to the development of IP. The ability to track the behaviour, hormone status, and physical condition of different hens and their progeny within commercial production environments would greatly extend current knowledge about predictors of IP in laying hens.

## Conclusions

**Figure 6:**
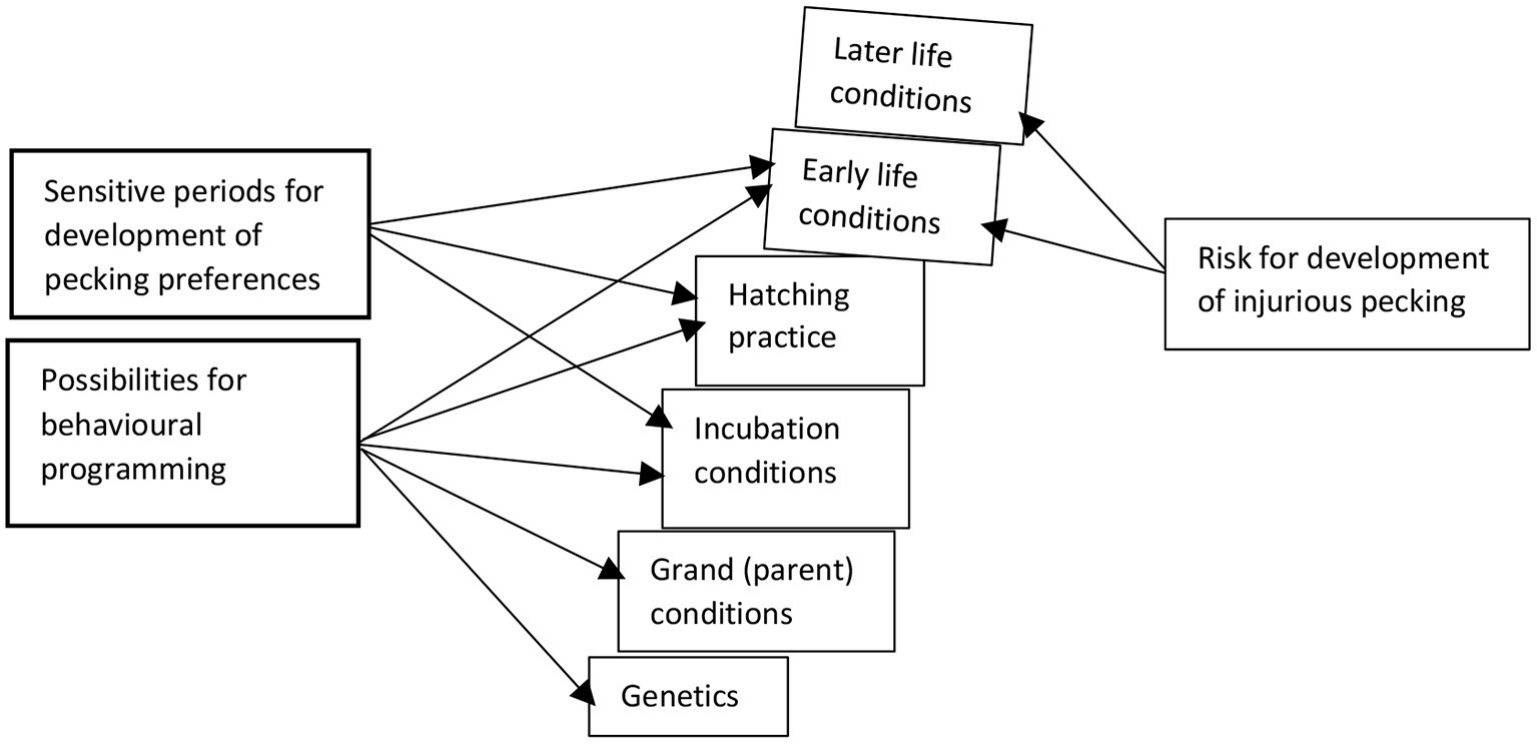
Jenga tower of risks for development of injurious pecking in laying hens, based on aspects in the production chain where possibilities for behavioural programming could occur and sensitive periods in early life could prime pecking preferences.

With this review, we have shown that prehatch and early post-hatch conditions can influence behavioural programming, with the potential to alter the likelihood of IP. IP is a multifactorial problem that appears more likely to occur when commercial incubation and rearing conditions deviate from the optimal, leading to long-lasting changes in the developing embryo/newly hatched bird. Assessing the risk for development of IP on a farm can be seen as a Jenga® tower (Figure 6), where blocks are built on top of each other. When the base of the tower is unstable, for example, due to adverse prenatal conditions, only minor perturbations in early or later life may be needed for the tower to fall and IP to occur. We conclude that matching positive environmental conditions of previous generations to the current generation, optimising incubation conditions, reducing stress at hatch, providing features such as dark brooders that compensate for absence of some functions of the maternal hen, ensuring ample foraging opportunities, and guiding appropriate learning for chicks (e.g., from older birds) are strategies likely to aid in preventing IP in commercial laying hen flocks.

## Author Contributions

All authors listed have made a substantial, direct and intellectual contribution to the work, and approved it for publication.

## Conflict of Interest

The authors declare that the research was conducted in the absence of any commercial or financial relationships that could be construed as a potential conflict of interest.

## Abbreviations

5-HT: serotonin
CORT: corticosterone
E2: estradiol
ED: embryonic day
FCM: fecal corticosterone metabolites
GFP: gentle feather pecking
HPA: hypothalamus–pituitary–adrenal
IP: injurious pecking
LDI: light during incubation
P: progesterone
RH: relative humidity
SFP: severe feather pecking
T: testosterone
woa: weeks of age
WL: White Leghorn.
